# Characteristics of lymphocyte subset alterations in COVID-19 patients with different levels of disease severity

**DOI:** 10.1186/s12985-022-01926-8

**Published:** 2022-11-19

**Authors:** Wei Dai, Aifang Zhong, Qinghua Qiao, Jian Wu, Weiwei Li, Qiuyue Wu, Hongjian Zhou, Shijie Qin, Weijun Jiang, Jing Zhang, Xinyi Xia

**Affiliations:** 1 Institute of Laboratory Medicine, Jinling Hospital, Nanjing University School of Medicine, Nanjing, 210002 Jiangsu China; 2 School of Animal Science and Food Engineering, Jinling Institute of Technology, Nanjing, 210038 Jiangsu China; 3 Department of Laboratory Medicine and Blood Transfusion, Wuhan Huoshenshan Hospital, Wuhan, 430100 Hubei China; 4 Medical Technical Support Division, the 904Th Hospital, Changzhou, 213003 Jiangsu China; 5 Medical and Technical Support Department, Pingdingshan Medical District, The 989Th Hospital of the PLA Joint Logistic Support Force,, Pingdingshan, 467000 Henan China; 6 School of Biomedical and Informatics, Nanjing Medical University, Nanjing, 211166 Jiangsu China

**Keywords:** COVID-19, Immune cells, Lymphocyte subsets, IL-6, scRNA-seq

## Abstract

**Background:**

Coronavirus disease 2019 (COVID-19) is a respiratory disorder caused by severe acute respiratory syndrome coronavirus 2 (SARS-CoV-2), which had rapidly spread all over the world and caused public health emergencies in the past two years. Although the diagnosis and treatment for COVID-19 have been well defined, the immune cell characteristics and the key lymphocytes subset alterations in COVID-19 patients have not been thoroughly investigated.

**Methods:**

The levels of immune cells including T cells, B cells, and natural killer (NK) cells in 548 hospitalized COVID-19 patients, and 30 types of lymphocyte subsets in 125 hospitalized COVID-19 patients admitted to Wuhan Huoshenshan Hospital of China were measured using flow cytometry. The relationship between lymphocytes subsets with the cytokine interleukin-6 (IL-6) and the characteristics of lymphocyte subsets in single-cell RNA sequencing (scRNA-seq) data obtained from peripheral blood mononuclear cells (PBMCs) were also analysed in COVID-19 patients.

**Results:**

In this study, we found that patients with critical COVID-19 infection exhibited an overall decline in lymphocytes including CD4^+^ T cells, CD8^+^ T cells, total T cells, B cells, and NK cells compared to mild and severe patients. However, the number of lymphocyte subsets, such as CD21^low^ CD38^low^ B cells, effector T4 cells, and PD1^+^ depleted T8 cells, was moderately increased in critical COVID-19 patients compared to mild cases. Notably, except for effector memory T4 cells, plasma blasts and Tregs, the number of all lymphocyte subsets was markedly decreased in COVID-19 patients with IL-6 levels over 30-fold higher than those in healthy cases. Moreover, scRNA-seq data showed obvious differences in the distribution and numbers of lymphocyte subsets between COVID-19 patients and healthy persons, and subsets-specific marker genes of lymphocyte subsets including CD4, CD19, CCR7, and IL7R, were markedly decreased in COVID-19 patients compared with those in healthy cases.

**Conclusion:**

A comprehensive decrease in immune cell and lymphocyte subsets in critical COVID-19 patients, and peripheral lymphocyte subset alterations showed a clear association with clinical characteristics.

**Supplementary Information:**

The online version contains supplementary material available at 10.1186/s12985-022-01926-8.

## Background

Coronavirus disease 2019 (COVID-19) is a distinct clade of the β-coronaviruses caused by severe acute respiratory syndrome coronavirus 2 (SARS-CoV-2), which has spread more than 160 countries and exerted a tremendous pressure on national health systems [1, 2]. Due to its global spread, the World Health Organization (WHO) declared COVID-19 as a public health emergency of international concern [3, 4]. Most patients infected with COVID-19 have mild illness and present common symptoms such as fever, dry cough, fatigue, and respiratory distress [5]. A small number of infected patients progress to severe cases with acute respiratory distress syndrome (ARDS); some severe patients even develop critical cases, worsen in a short period of time and eventually die of multiple organ failure [6]. It has been reported that COVID-19 is more likely to occur in elderly male persons with comorbidities [7]. Scientists and clinicians worldwide have made great efforts to explore specific and effective antiviral drugs [8, 9] and vaccines for COVID-19 [10, 11]. However, to date, there has been little thorough investigation about the immune response and lymphocyte subset changes in COVID-19 patients [12, 13].

The dysregulation of the immune system, such as lymphopenia and the so-called cytokine storm, has been commonly observed in COVID-19 patients, it is believed to be associated with the severity of pathogenic coronavirus infections and the exacerbation of lung damage similar to Middle East respiratory syndrome coronavirus (MERS-CoV) infections [14, 15]. It is known that immune cell and lymphocyte subsets of CD4^+^ T cells, CD8^+^ T cells, B cells, and NK cells play an important role in the maintenance of immune system function. Recent studies have highlighted a reduction in the numbers of lymphocytes and their subsets, particularly CD4^+^ T cells and CD8^+^ T cells, and an increase in inflammatory cytokine levels in peripheral blood after COVID-19 infection [16]. It has also been showed that the immune system play a crucial function in response to COVID-19 infection with significant differences among severe and non-severe patients [17]. However, the key immune cell subset changes and their states during COVID-19 infection remain largely unclear. Thus, examination of key lymphocyte subsets severe and non-severe COVID-19 patients is a crucial step in identifying patients with the risk of unfavorable course of this disease, predicting the prognosis and recognizing improvement in the clinical status [18]. Moreover, detailed analysis of lymphocyte subset alternations could also help to develop novel prospective therapeutic strategies for COVID-19 [19].

Here, in this study, immune cells including T cells, B cells, NK cells, from 548 hospitalized COVID-19 patients and 30 types of lymphocyte subsets, such as transitional B cells, central memory T4 cells, effector T8 cells, mature NK cells, and Tregs, from 125 hospitalized COVID-19 patients admitted to Wuhan Huoshenshan Hospital of China were measured using flow cytometry. The relationship between proinflammatory cytokine IL-6 levels and T, B, NK, and Treg lymphocyte subsets was also analysed. We also applied single-cell RNA sequencing (scRNA-seq) data to comprehensively describe the characteristics of lymphocyte subsets in peripheral blood mononuclear cells (PBMCs) of COVID-19 patients compared to healthy persons.

## Methods

### Experimental design and patients

This study was approved by the Medical Ethical Committee of Wuhan Huoshenshan Hospital of China. Written informed consent was obtained from each patient. A total of 548 individuals with COVID-19 infection admitted to Wuhan Huoshenshan Hospital from February 4 to April 12, 2020 were enrolled in this study, and measured with T cell, B cell, and NK cell using flow cytometry, including 194 individuals (95 [48.97%] males and 99 [51.03%] females; median age 58.0 years [IQR 49.0‒65.0] had mild COVID-19 symptoms, 304 individuals (148 [48.68%] males and 156 [51.32%] females; median age 64.0 years [IQR 55.0‒71.0] were diagnosed with severe COVID-19 symptoms, and 50 individuals (34 [68.0%] males and 16 [32%] females; median age 71.0 years [IQR 65.0‒81.0]) were diagnosed with critical COVID-19 symptoms. In addition, a total of 125 patients with COVID-19 infection admitted to Wuhan Huoshenshan Hospital were measured with 30 types of lymphocyte subsets, including 33 individuals (17 [51.5%] males and 16 [48.5%] females; median age 63.0 years [IQR 50.0‒68.0]) had mild COVID-19 symptoms, 75 individuals (47 [62.7%] males and 28 [37.3%] females; median age 67.0 years [IQR 58.0‒74.0]) were diagnosed with severe COVID-19 symptoms, and 17 individuals (11 [64.7%] males and 6 [35.3%] females; median age 75.0 years [IQR 67.0‒81.0]) were diagnosed with critical COVID-19 symptoms. All patients were confirmed by nucleic acid tests in throat swab samples using a standard SARS-CoV-2 nucleic acid detection kit.

### Data collection

The information of each patient was extracted from electronic medical records, including age, sex, medical history, symptoms, clinical classification on admission, laboratory findings, treatment, and efficacy. The diagnosis of COVID-19 infection and clinical classification was determined according to clinical classification criterion in the Diagnosis and Treatment Protocol for Novel Coronavirus Pneumonia (7^th^ trial version), issued by the National Health Committee of the People's Republic of China [20]. In addition, the criteria that can potentially affect the immune status of the patients, including rheumatic diseases, systemic lupus erythematosus, scleroderma, idiopathic thrombocytopenic purpura, autoimmune processes, and immunodeficiencies, was excluded in this study. On admission, severe illness was defined to meet the following criteria: (1) respiratory distress with a respiratory rate > 30 breaths/min, (2) oxygen saturation ≤ 93% in the resting state, and (3) the ratio of partial pressure of arterial oxygen (PaO_2_) to the fraction of inspired oxygen (FiO_2_) ≤ 300 mmHg.

### Flow cytometry assay

Peripheral blood was collected from patients with COVID-19 infection who were admitted to Wuhan Huoshenshan Hospital, and all samples were tested within six hours after being obtained. Briefly, 100 μL of fresh whole blood was incubated in 2 mL of VersaLyse (Beckman Coulter Life Science) between 20 ℃ and 30 ℃ for 15 min to lyse erythrocytes and then washed with 3 mL of 1 × PBS. Thereafter, the cell pellet was resuspended in 500 μL of 1 × PBS containing 0.8% IOTest 3 fixative solution. The samples were then ready for acquisition. To measure T cell, B cell, NK cell and lymphocyte subsets, we stained all ten single colour tubes from a single pouch of the Compensation Kit provided in the IM DuraClone IM cell subsets Tube, 25 tests (Table 1), RUO with venous blood, measured by multiple-colour 13 DxFLEX3 Flow Cytometer according to the manufacturer’s instructions (Backman Coulter Life Science). The data were evaluated using the Beckman Kaluza Software.

**Table 1:** The information of DuraClone IM T cell subsets Tube

λ Excitation	405 nm		488 nm					633 nm				
Product	PB	KrO	FITC	PE	ECD	PC5.5	PC7	APC	AF647	AF700	APC-AF700	APC-AF750
Phenotyping basic	–	CD45	CD16	CD56	CD19	–	CD14	CD4	–	CD8	–	CD3
B cell	IgM	CD45	IgD	CD21	CD19	–	CD27	CD24	–	–	–	CD38
T cell subsets	CD57	CD45	CD45RA	CCR7	CD28	PD1	CD27	CD4	–	CD8	–	CD3
Dendritic cells	HLA–DR	CD45	CD16	*	–	CD1c	CD11c	Clec9A	–	–	CD123	–
TCRs	TCRVδ2	CD45	TCRγδ	TCRαβ	HLA–DR	–	TCRVδ1	CD4	–	CD8	–	CD3
Treg	Helios	CD45	CD45RA	CD25	–	CD39	CD4	–	FoxP3	–	–	CD3
Granulocytes	CD15	CD45	CD294	–	CD16	CD33	CD11b	PD–L1		–	**	CD62L
Count	–	–	CD45	Counting beads		7–AAD	–	–		–	–	–

*PB* Pacific Blue; *KrO* Krome Orange; *FITC* Fluorescein isothiocyanate; *PE* Phycoerythrin; *ECD* Phycoerythrin Texas Red-X; *PC5.5* Phycoerythrin Cyanine 5.5; *PC7* Phycoerythrin Cyanine 7; *APC* Allophycocyanin; *AF647* Alexa Fluor 647; *AF700* Alexa Fluor 700; *APC-AF700* Allophycocyanin Alexa Fluor 700; *APC-AF750* Allophycocyanin Alexa Fluor 750

*CD3/CD19/CD20/CD14/CD56; **, CD3/CD14/CD19/CD56

### scRNA-seq data

The single-cell RNA sequencing (scRNA-seq) data in peripheral blood mononuclear cells (PBMCs) were downloaded from NCBI Gene Expression Omnibus (accession no. GSE150728) [21], in which data from three COVID-19 patients and three healthy persons data were used in this study. The distinct subsets of lymphocyte subsets were subclustered using Uniform Manifold Approximation and Projection (UMAP). The R package was used for data analysis and visualization.

### Statistical analysis

The data are indicated as the means ± standard deviations (SD) and were measured by GraphPad Prism 8.0. The statistical significance was analysed by Kruskal‒Wallis One-way ANOVA nonparametric test. Differences at *p* < 0.05 were considered statistically significant.

## Results

### An overview of T, B, and NK cells in COVID-19 patients

**Fig. 1:**
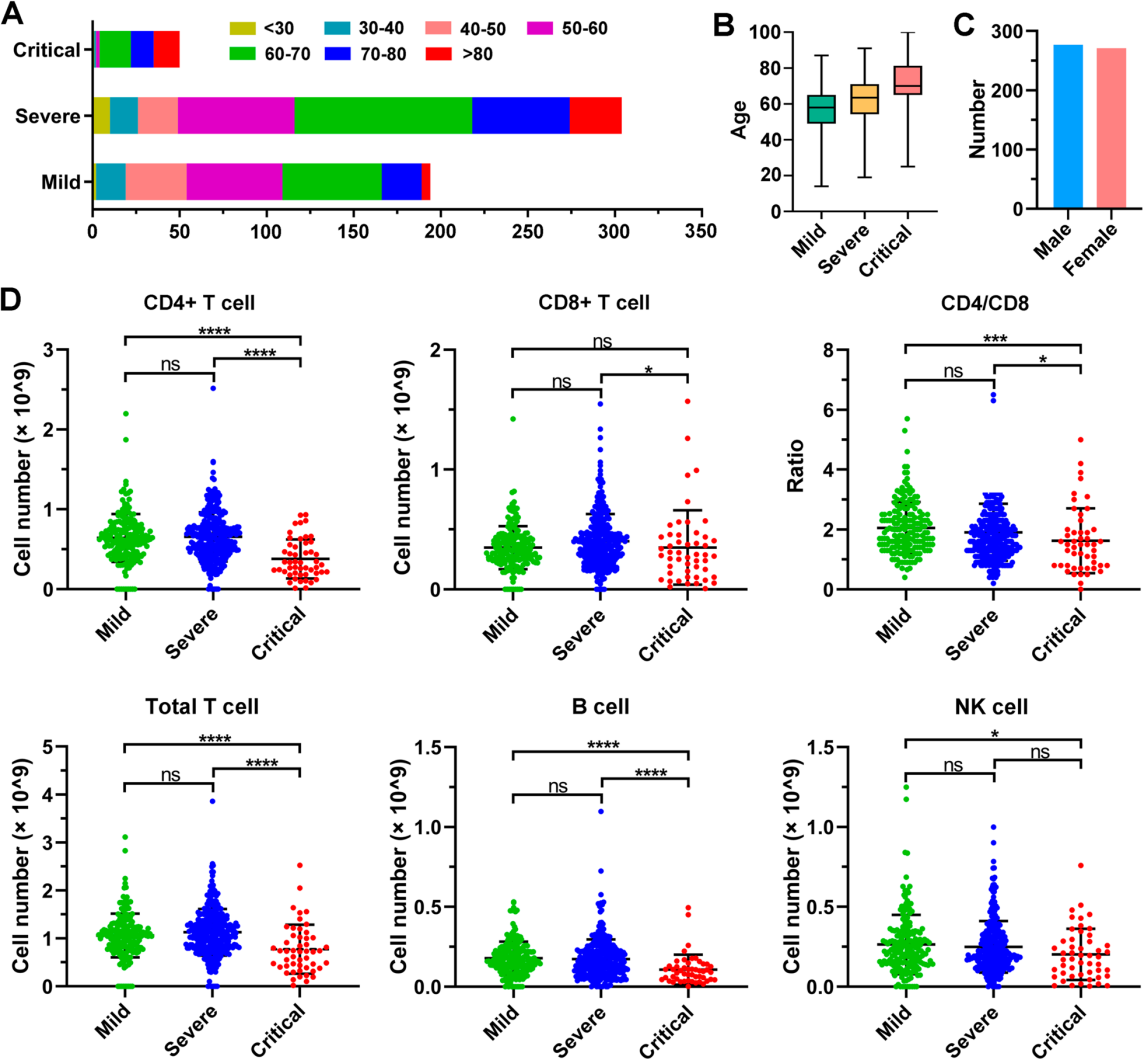
The number of T, B, and NK lymphocytes in COVID-19 patients at hospital admission. **A** The distribution of ages in mild, severe and critical COVID-19 patients. **B** Boxplot showing the mild, severe and critical COVID-19 patients’ age as Median and Interquartile range (IQR). **C** The distribution of sex in 548 COVID-19 patients. **D** The number of T, B, and NK lymphocyte cells in 548 COVID-19 patients. ns, nonsignificant; *p < 0.05; ***p < 0.001; ****p < 0.0001. Kruskal‒Wallis test for comparing the mean difference

Peripheral blood was collected from 548 individuals with COVID-19 infection at inpatient admission to Wuhan Huoshenshan Hospital, and T cells, B cells, and NK cells were measured using flow cytometry. Of these patients, 35.4% (194/548) had mild COVID-19 symptoms, 55.5% (304/548) were diagnosed with severe COVID-19 symptoms, and 9.1% (50/548) were diagnosed with critical COVID-19 symptoms. A total of 72.2% (140/194) of mild COVID-19 patients, 83.9% (255/304) of severe patients, and 96% (48/50) of critical patients were over 50 years old (Fig. 1A). We found that only 2.6% (5/194) of mild COVID-19 patients and 9.9% (30/304) of patients with severe illness were over 80 years old, while up to 30% (15/50) of critical patients were more than 80 years old (Fig. 1A). A total of 277 (50.5%) males and 271 (49.5%) females with COVID-19 infection were analysed in this experiment (Fig. 1B).

As indicated in Fig. 1C, unlike CD8 T cells, the ratio of CD4 to CD8 (CD4/CD8), CD4 + T cells, and total T-cell numbers in COVID-19 critical patients were significantly decreased (*p* < 0.001) compared to mild cases. CD4 T-cell and total T-cell numbers in critical COVID-19 patients were significantly lower than those in severe patients (*p* < 0.0001), and CD8 T-cell numbers in critical COVID-19 patients were also markedly decreased compared to those in severe patients (*p* < 0.05). However, CD4 T cell, CD8 T cell, and total T-cell numbers were not remarkably changed in severe patients compared to mild COVID-19 patients (*p* > 0.05). Notably, the CD4 T-cell to CD8 T-cell ratio, consistent with the change in lymphocytes, was significantly lower in critical patients than in mild cases (*p* < 0.001), and also lower in critical patients than in severe patients (*p* < 0.05), suggesting that the CD4 to CD8 T-cell ratio is associated with disease severity (Fig. 1C). Similarly, compared to mild COVID-19 patients, the B cell number was significantly decreased in severe patients (*p* < 0.0001), while critical patients had significantly lower B-cell numbers than severe patients (*p* < 0.0001). In addition, the NK-cell number was not significantly different in severe patients compared to mild patients, or in critical illness patients compared to severe patients (*p* > 0.05), though it was significantly decreased in critical patients in comparison with mild patients (*p* < 0.05) (Fig. 1C).

### B-cell subset numbers are reduced in COVID-19 severe/critical patients

**Fig. 2:**
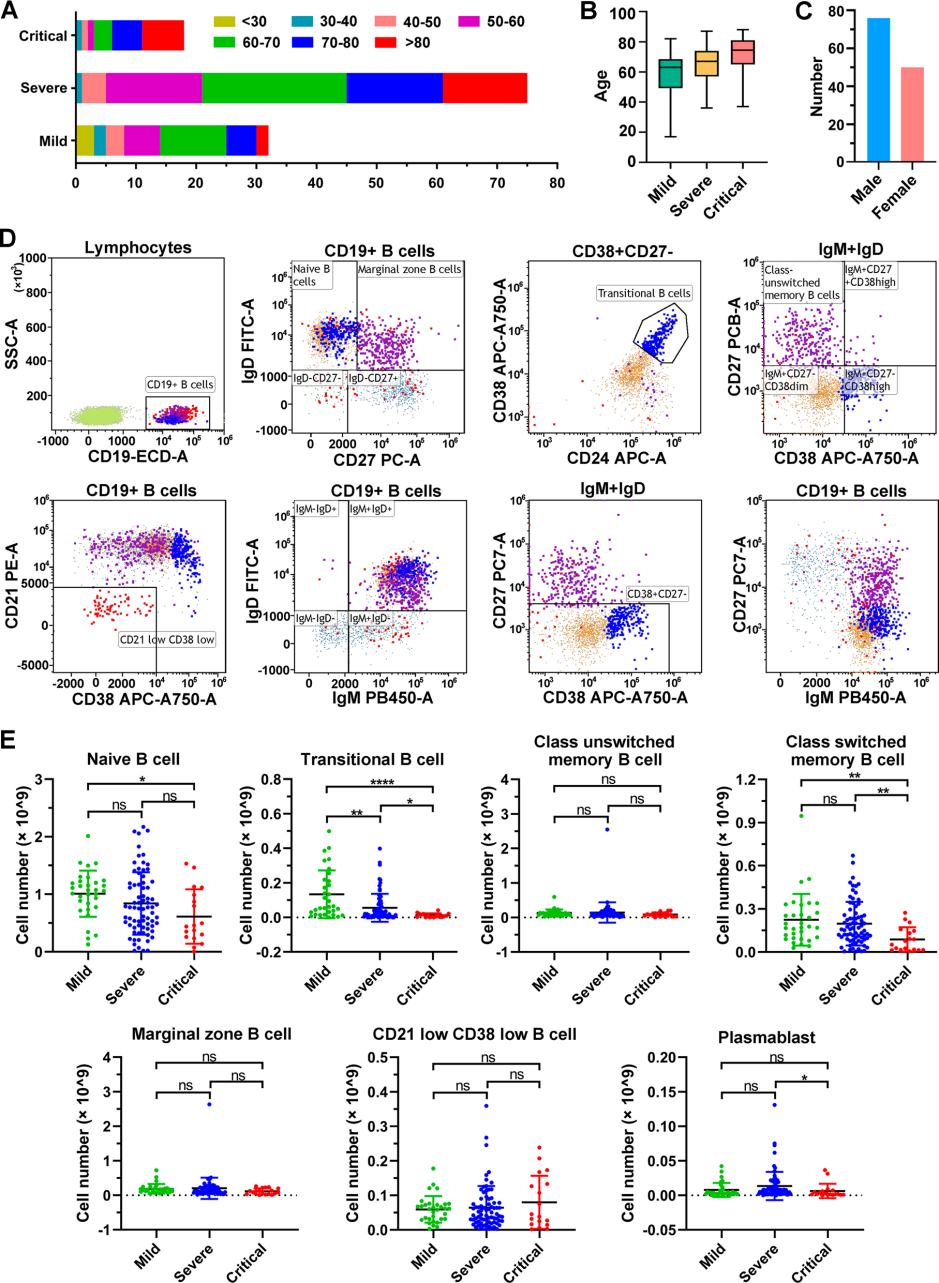
The number of B lymphocyte subsets in COVID-19 patients at hospital admission. **A** The distribution of ages in mild, severe and critical COVID-19 patients. **B** Boxplot showing the mild, severe and critical COVID-19 patients' age as Median and Interquartile range (IQR). **C** The distribution of sex in 126 COVID-19 patients. **D** Representative flow cytometry dot plots showing the gating strategy for B-cell subsets. **E** The number of B lymphocyte subsets in 126 COVID-19 patients. ns, nonsignificant; *p < 0.05; **p < 0.01; ****p < 0.0001. Kruskal‒Wallis test for comparing the mean difference

To investigate the lymphocyte subset alterations in COVID-19 patients, peripheral blood was collected from 125 individuals with COVID-19 infection admitted to Wuhan Huoshenshan Hospital, of whom 33 patients had mild COVID-19 symptoms, 75 individuals were diagnosed with severe COVID-19 symptoms, and 17 individuals were diagnosed to present critical COVID-19 symptoms. As shown in Fig. 2A, 54.5% (18/33) of mild COVID-19 patients were more than 60 years old, 72% (54/75) patients with of severe COVID-19 illness were over 60 years old, while only three (16.7%) critical COVID-19 patients were under 60 years old, and 38.9% (7/18) of illness were over 80 years old (Fig. 2A). A total of 75 (60.0%) male and 50 (40.0%) female were evaluated in this experiment (Fig. 2B).

B lymphocyte subsets including naïve B cells, transitional B cells, class switched/unswitched memory B cells, marginal zone B cells, CD21^low^ CD38^low^ B cells, and plasma blasts, were evaluated using flow cytometry with a representative gating strategy (Fig. 2C, Fig. S1). Figure 2D shows that compared to mild COVID-19 patients, naïve B cells, transitional B cells, and class-switched memory B-cell numbers were markedly decreased in critical patients (*p* < 0.05) (Fig. 2D). The transitional B-cell count, class-switched memory B-cell count, and plasma blast number were significantly lower in critical COVID-19 patients than those in patients with severe illness (*p* < 0.05). Notably, the CD21 low CD38 low B cell number was moderately increased in severe and critical COVID-19 patients than those in mild cases. There was no significant difference of class unswitched memory B cell, marginal zone B cell, CD21 low CD38 low B cell number was observed between mild, severe and critical COVID-19 patients (*p* > 0.05) (Fig. 2D). Notably, only the transitional B-cell number was significantly decreased in severe COVID-19 patients compared to mild patients (*p* < 0.01), and significantly lower in critical patients than in severe patients (*p* < 0.05) (Fig. 2D), indicating that transitional B-cell may be associated with disease severity.

### T-cell subset alterations in COVID-19 patients

**Fig. 3:**
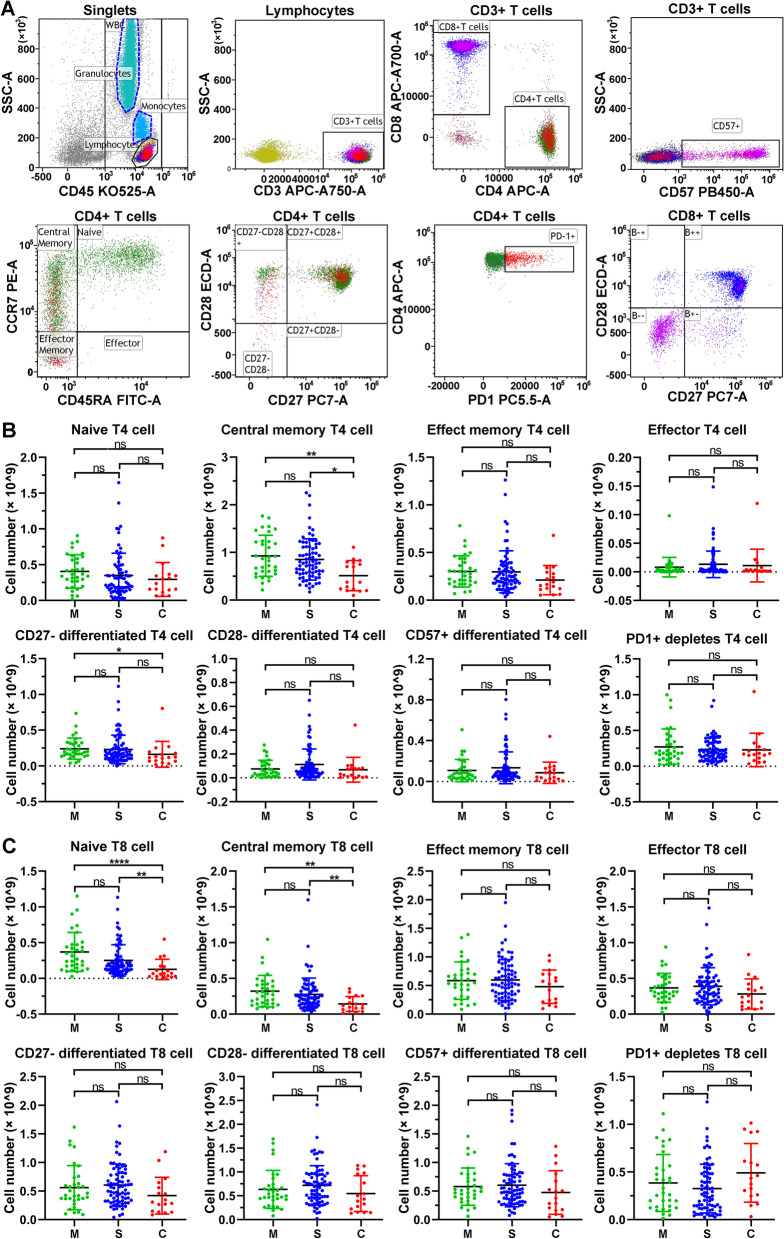
The number of T lymphocyte subsets in COVID-19 patients at hospital admission. **A** Representative flow cytometry dot plots showing the gating strategy for T4 and T8 lymphocyte subsets. **B** The number of T4 lymphocyte subsets in 126 COVID-19 patients. **C** The number of T8 lymphocyte subsets in 126 COVID-19 patients. *ns* nonsignificant; *p < 0.05; **p < 0.01. Kruskal‒Wallis test for comparing the mean difference

To analyse the adaptive immune cell populations, T lymphocyte subsets, including naïve T4/T8 cell, central memory T4/T8 cells, effect memory T4/T8 cells, effector T4/T8 cells, CD27^−^ differentiated T4/T8 cells, CD28^−^ differentiated T4/T8 cells, CD57^+^ differentiated T4/T8 cells, and PD1^+^ depleted T4/T8 cells, were analysed using flow cytometry with a representative gating strategy (Fig. 3A, Fig.S2). The central memory T4 cell number was significantly decreased in critical COVID-19 patients compared to mild and severe patients (*p* < 0.05), but there was no significant difference observed between mild and severe patients (Fig. 3B). The CD27^−^ differentiated T4 cell number in critical COVID-19 patients was also obviously lower than that in patients with mild illness (*p* < 0.05), while there was no significant change between mild and severe patients or between neither did severe and critical patients (*p* > 0.05). Moreover, there was also no significant difference in other T4 lymphocytes, such as naïve T4 cells, effect memory T4 cells, effector T4 cells, CD28^−^ differentiated T4 cells, CD57^+^ differentiated T4 cells, and PD1^+^ depleted T4 cells, between mild, severe and critical COVID-19 patients (Fig. 3B). Similarly, the central memory T8 cell number was decreased in COVID-19 severe patients, and significantly reduced in critical patients, compared to mild patients (*p* < 0.01), but no significant difference was observed between mild and severe patients (Fig. 3C). The naïve T8 cell number in critical COVID-19 illness was significantly lower than that in mild patients (*p* < 0.0001), and significantly decreased compared with that in severe patients (*p* < 0.01), but no significant difference was found between mild and severe patients (*p* > 0.05) (Fig. 3C). In addition, there no significant difference in other T8 lymphocyte subsets, including effector memory T8 cells, effector T8 cells, CD27^−^ differentiated T8 cells, CD28^−^ differentiated T8 cells, CD57^+^ differentiated T8 cells, and PD1^+^ depleted T8 cells, between mild, severe and critical COVID-19 patients (Fig. 3C).

### NK-cell subsets and Treg cells are decreased in COVID-19 severe/critical patients

**Fig. 4:**
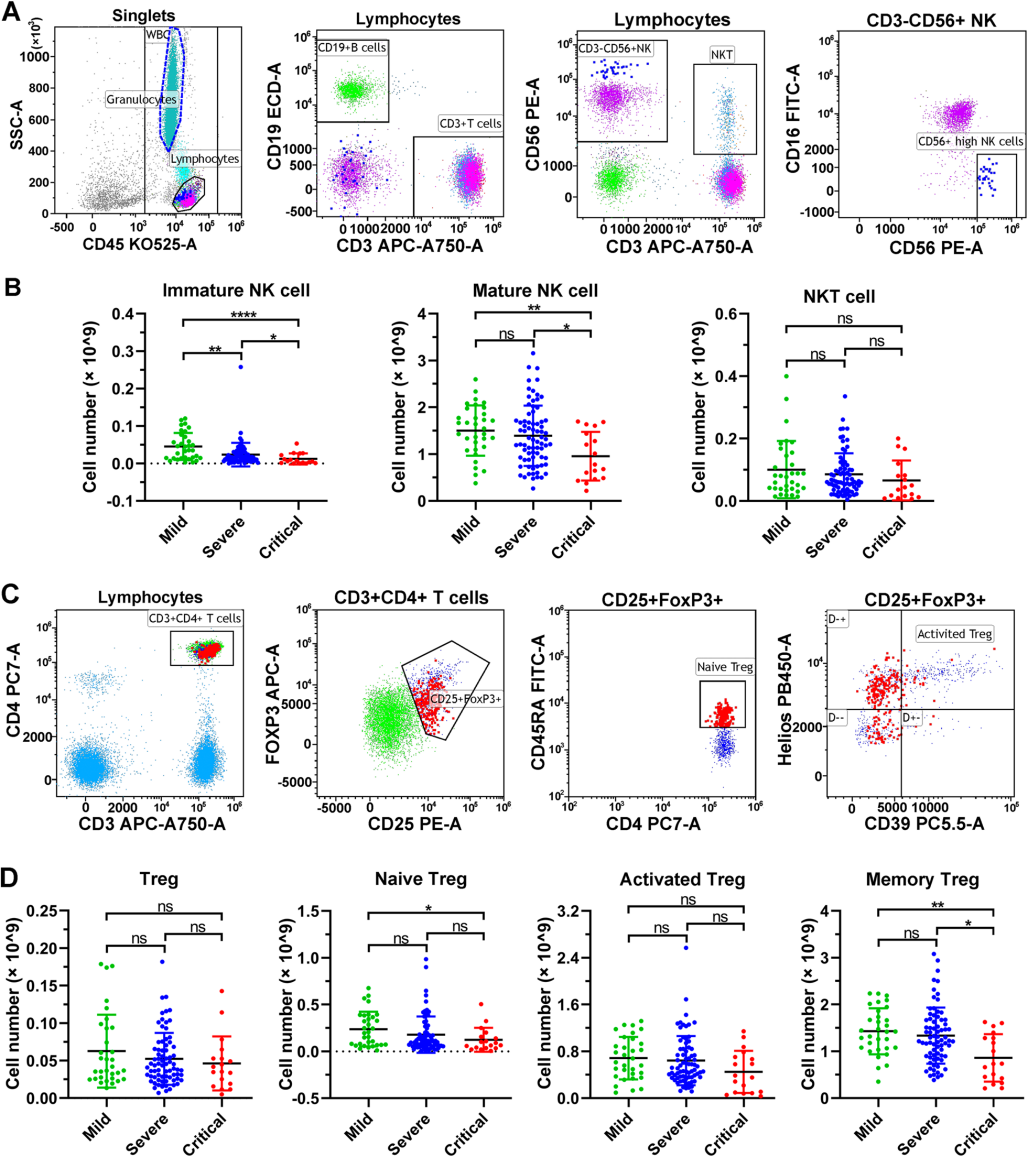
The number of NK and Treg lymphocyte subsets in COVID-19 patients at hospital admission. **A** Representative flow cytometry dot plots showing the gating strategy for NK-cell subsets. **B** The number of NK lymphocyte subsets in 126 COVID-19 patients. **C** Representative flow cytometry dot plots showing the gating strategy for Treg subsets. **D** The number of NK lymphocyte subsets in 126 COVID-19 patients. ns, nonsignificant; *p < 0.05; **p < 0.01; ****p < 0.0001. Kruskal‒Wallis test for comparing the mean difference

Other immune cells including immature NK-cells, mature NK-cells, and CD3^+^CD16^+^CD56^+^ NKT cells, were also evaluated in blood samples (Fig. 4A, Fig. S3). We found that in comparison with the mild COVID-19 patients, there was an obvious trend of a decrease in immature NK-cell number in severe patients (*p* < 0.01), and a notable reduction in critical patients (*p* < 0.0001). There was also a significant decrease between severe and critical patients (*p* < 0.05), indicating that immature NK-cells are associated with disease severity (Fig. 4B). A similar decrease was also observed in mature NK-cell number in critical COVID-19 illness compared to mild and severe patients (*p* < 0.05), but no obvious difference was observed in severe patients (Fig. 4B). No significant difference in the NKT-cell number was observed between mild, severe and critical COVID-19 patients (*p* > 0.05) (Fig. 4B).

Regulatory T cells (Tregs) are an important T-cell subset for immune tolerance and anti-inflammatory reactions. In this study, the numbers of Treg, naïve Treg, activated Treg, and memory Treg cells that suggestive for Treg-enriched population were measured by flow cytometry with representative gating strategy (Fig. 4C, Fig. S4). The number of naïve Treg cells was mildly decreased in severe COVID-19 patients, and significantly reduced in critical patients compared with patients with mild illness (*p* < 0.05) (Fig. 4D). There was a trend of a moderate decrease in the number of memory Treg cells in severe patients (*p* > 0.05), and a significant reduction in critical patients (*p* < 0.01) compared to mild patients, and an obvious decreasing trend between severe and critical patients (*p* < 0.05) (Fig. 4D). However, the number of Treg and activated Treg cells was not significantly different in mild, severe and critical COVID-19 patients (*p* < 0.05) (Fig. 4D).

### Relationship between IL-6 levels and lymphocyte subsets in COVID-19 patients with different severity

**Fig. 5:**
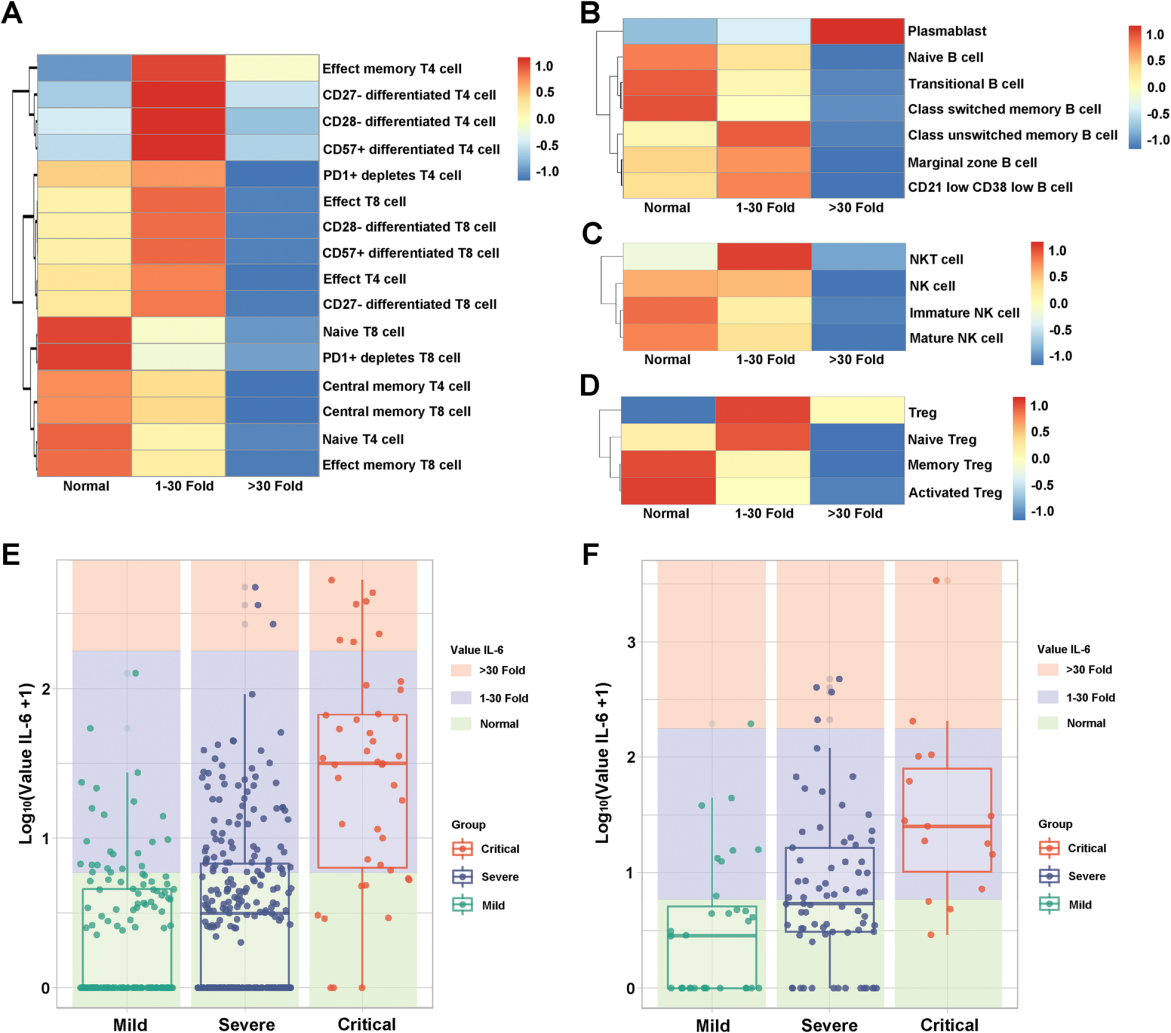
Heatmap showing the relationship between IL-6 levels and lymphocyte subsets in COVID-19 patients. **A** Heatmap showing the relationship between IL-6 levels and T lymphocyte subsets. **B** Heatmap showing the relationship between IL-6 levels and B lymphocyte subsets. **C** Heatmap showing the relationship between IL-6 levels and NK lymphocyte subsets. **D** Heatmap showing the relationship between IL-6 levels and Treg lymphocyte subsets. Normal, the IL-6 level was normal; 1–30-fold, the IL-6 level was 1–30-fold than normal; > 30-fold, the IL-6 level was above 30-fold higher than normal. **E** The relationship between IL-6 levels and 548 COVID-19 patients with different severity measured with T cells, B cells, and NK cells. **F** The relationship between IL-6 levels and 125 COVID-19 patients with different severity measured with lymphocyte subsets

Cytokines are crucial biomarkers of the progression of various inflammatory disorders including pneumonia. We characterized the relationship between the proinflammatory cytokine IL-6 and T, B, NK, and Treg lymphocyte subsets in the blood samples of 125 COVID-19 patients at inpatient admission. The number of T lymphocyte subsets, including naïve T4/T8 cells, central memory T4/T8 cells, PD1^+^ depletes T8 cells, and effector memory T8 cells, was moderately increased in COVID-19 patients with normal IL-6 levels, and with increasing IL-6 levels, these lymphocyte subset counts declined (Fig. 5A). The other T lymphocyte subset counts showed a decreasing trend with increasing IL-6 levels (Fig. 5A). In B lymphocyte subsets, it is worth noting that the number of plasma blasts was markedly increased in COVID-19 patients, with IL-6 levels above 30-fold higher than normal (Fig. 5B), but other B lymphocyte subsets, including naïve B cells, transitional B cells, class switched memory B cells, class unswitched memory B cells, marginal zone B cells, and CD21^low^ CD38^low^ B cells, were obviously decreased with higher IL-6 levels (Fig. 5B). As demonstrated in Fig. 5C, the higher IL-6 the levels in COVID-19 patients, the lower the number of NK lymphocyte subsets, including NKT cells, NK cells, immature NK cells, and mature NK cells (Fig. 5C). Similarly, the numbers of naïve Tregs, memory Tregs and activated Tregs were significantly decreased with higher IL-6 levels, but when the level of IL-6 was 30-fold higher than normal, Tregs were instead increased compared to COVID-19 patients with normal IL-6 levels (Fig. 5D).

Further, we also investigate the relationship between the IL-6 and COVID-19 patients with the different levels of disease severity in 584 individuals measured with T cells, B cells, NK cells, and 125 individuals measured with lymphocyte subsets, respectively. In 584 COVID-19 patients, the IL-6 levels showed an increasing trend with increasing disease severity, it is worth noting that the IL-6 values were concentrating in normal level in mild COVID-19 patients, while it was markedly increasing in critical patients (Fig. 5E). Similarly, in 125 COVID-19 patients measured with lymphocyte subsets, the IL-6 levels were significantly increased with increasing disease severity, with the median IL-6 values in critical COVID-19 patients were significant higher than those of mild patients (Fig. 5F). It was found that as a crucial cytokine, IL-6 itself could also mark the disease severity of COVID-19.

### Characteristics of lymphocyte subsets in COVID-19 patients with scRNA-seq

**Fig. 6:**
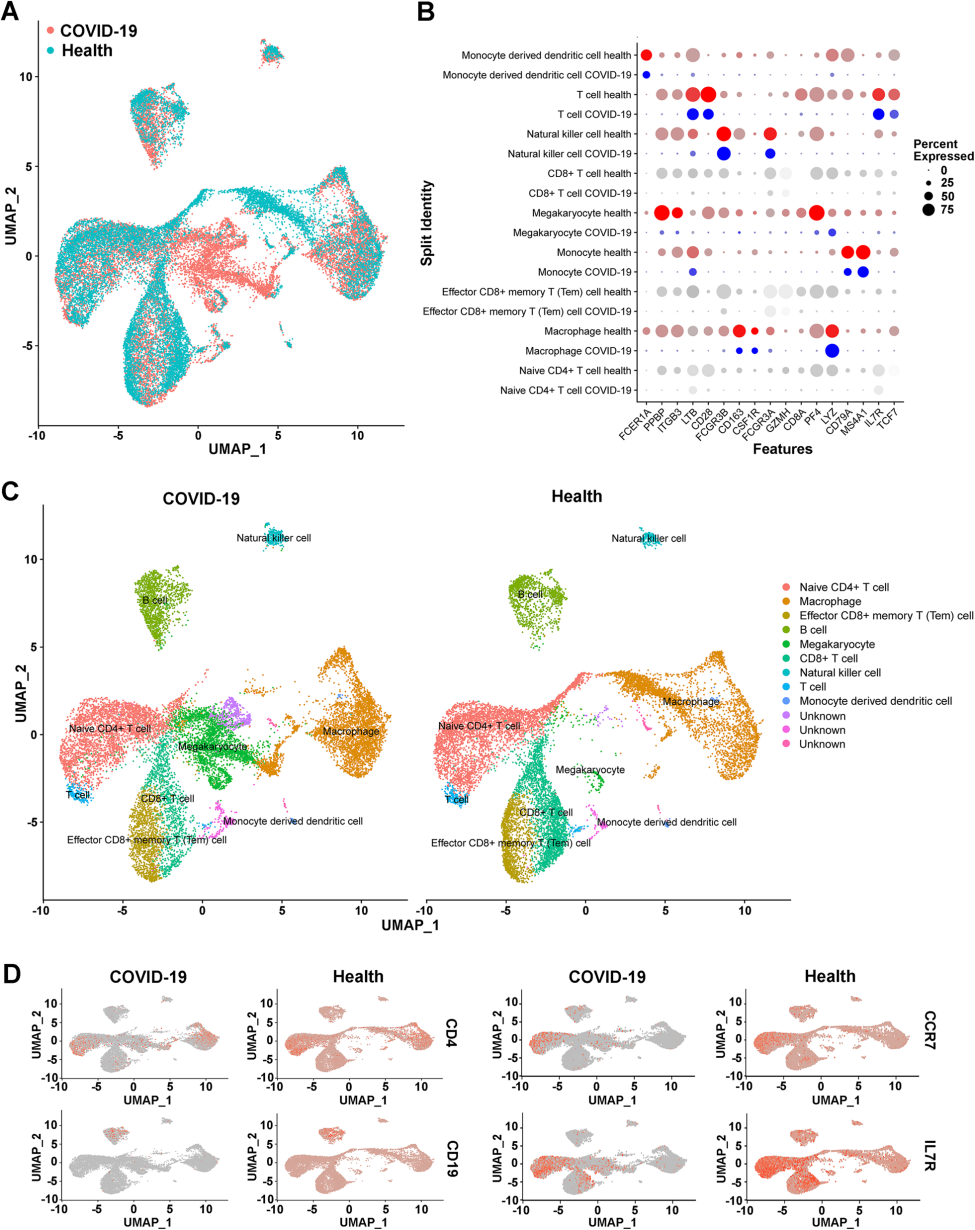
Characteristics of lymphocyte subsets between COVID-19 patients and healthy controls. **A** The UMAP plots show the difference in lymphocyte distribution. **B** Comparison of high-expression genes in lymphocyte subsets. **C** UMAP plot shows the nine subclusters of T, B, and NK lymphocytes in COVID-19 patients and healthy controls. **D** UAMP plot shows subset-specific marker genes of lymphocytes between COVID-19 patients and healthy controls

To explore the characteristics of T cell, B cell, NK-cell and Treg lymphocyte subsets in COVID-19 patients compared to healthy individuals, we also used scRNA-seq data in this study. The uniform manifold approximation and projection (UMAP) plots indicated significant differences in lymphocyte counts between COVID-19 patients and healthy persons, and overall lymphopenia was found in patients with COVID-19 infection (Fig. 6A). The expression of cell-specific genes such as FCER1A in monocyte-derived dendritic cells, PPBP in megakaryocyte, ITGB3 in NK cells, and PF4 in T cells of COVID-19 patients was obviously lower than that in healthy controls (Fig. 6B). As demonstrated in Fig. 6C, UMAP plots showed the 9 subclusters of T cell, B cell, and NK lymphocyte subsets, including naïve CD4^+^ cells, macrophages, effector memory (TEM) CD8^+^ T cells, B cells, megakaryocytes, CD8^+^ cells, natural killer cells, T cell, and monocyte-derived dendritic cells in COVID-19 patients and healthy controls, and three unknown subclusters were also identified (Fig. 6C). Moreover, four lymphocyte subset-specific marker genes in blood including CD4 in Tregs, activated CD4^+^ T cells, naïve CD4^+^ T cells, T helper cells, CD19 in memory B cells, activated CD4^+^ T cells, immature transitional B cells, naïve B cells, NK cells, CCR7 in effector memory CD4^+^ T cells, naïve T cells, and IL7R in Tregs were significantly decreased in COVID-19 patients compared to healthy controls (Fig. 6D).

## Discussion

The rapid spread of COVID-19 has induced an urgent demand for understanding the role of immunity in the progression of viral infection and subsequent pneumonia. As a new member of the coronavirus family, infection with COVID-19 can cause a cytokine storm, leading to a high incidence of immune disorders [22]. It is known that lymphocytes and their subsets play important roles in the maintenance of immune system function [23]. However, until recently, very few publications have characterized the comprehensive changes in lymphocytes in COVID-19-infected individuals.

As with immune diseases and viral infections, such as MERS-CoV and influenza infections [14, 15], it is generally accepted that host immune responses determine both protection against viral infections and the pathogenesis of respiratory injury, and can lead to dysregulation in the levels of lymphocyte subsets [24, 25]. Cellular surface molecules such as CD3^+^CD4^+^ mark T helper cells (Th cells), CD3^+^CD8^+^ mark cytotoxic T lymphocytes (CTLs), CD19^+^ mark B cells, and CD16^+^CD56^+^ mark NK cells. These cells are also involved in humoral and cytotoxic immunity against viral infection. Thus, it is important to clarify the characteristics of lymphocyte subsets in COVID-19 patients, in order to provide novel insights of the immune mechanism.

In this study, we analysed almost all immune cell types and lymphocyte subsets in peripheral blood collected from mild, severe and critical COVID-19 patients admitted to Wuhan Huoshenshan Hospital. We found that lymphopenia was common in COVID-19 patients, which might be due to the recruitment of reactive lymphocytes in the lungs and an impairment of the immune system during the progression of COVID-19. Significant decreases in CD4^+^ T cells, total T cells, and B cells were observed in critical COVID-19 patients. No significant differences in CD4^+^ T cells, CD8^+^ T cells, total T cells, B cells, or NK cells were found between COVID-19 mild and severe patients. Cui et al. reported that the incidence of lymphopenia in SARS patients was 84%, CD4^+^ T cells decreased 100%, CD8^+^ T cells decreased 87%, B cells decreased 76%, and NK cells decreased 55% [26]. In a study by Assiri et al. on MERS, lymphopenia occurred in 34% of patients [14]. Lymphopenia may be caused by virus attachment or indirectly by immune injuries from inflammatory mediators. Moreover, exudation of circulating lymphocytes into inflammatory lung tissues may also lead to lymphopenia. In addition, we also found that CD4^+^ T cells and CD8^+^ T cells in COVID-19 critical cases showed greater reductions than those in severe patients. This phenomenon suggests that T lymphocytes play an important immune defence function against COVID-19 infection.

The lymphocyte subsets of CD4^+^ T cells, CD8^+^ T cells, B cells and NK cells were also analysed in the peripheral blood of COVID-19 inpatients in this study. Overall lymphopenia of subsets was common in critical COVID-19 cases compared to mild patients, which may indicate serious conditions and a higher mortality rate of critical cases. The reduction in the T-cell subset number was more pronounced in CD8^+^ T-cell subsets than in CD4^+^ T-cell subsets. Instead, the number of effector T4 cells and PD1^+^ depleted T8 cells in critical COVID-19 patients, and effector T4 cells, CD28^−^ differentiated T4 cells, CD57^+^ differentiated T4 cells, effector memory T8 cells, effector T8 cells, CD27^−^ differentiated T8 cells, CD28^−^ differentiated T8 cells, and CD57^+^ differentiated T8 cell subsets in severe patients were enlarged compared to mild cases, possibly indicating an active anti-inflammatory response. Despite the reduction in total absolute of B cells and CD21^low^ CD38^low^ B cells in severe and critical COVID-19 patients, plasma blasts in severe patients were still increased. Moreover, it is interesting that regulator T cells (Tregs) and natural killer T (NKT) cells, which were necessary for immunoregulation and anti-inflammatory response [27], and correlated with disease progression [28, 29], were moderately decreased in severe and critical COVID-19 patients, suggesting possible immunosuppression.

As COVID-19 infection has been reported to be associated with increased IL-6 levels [30], we evaluated the relationship between this proinflammatory cytokine in the blood with T, B, NK, and Treg lymphocyte subsets in this study. There are three classifications of the relationship between IL-6 levels and lymphocyte subsets. With increasing the IL-6 levels, most lymphocyte subsets decreased due to the inflammatory response, such as naïve T8 cells, transitional B cells, immature NK cells, and activated Tregs. A small portion of lymphocyte subsets, such as effect T8 cells, class unswitched memory B cells, and naïve Tregs, were increased during initial inflammatory progression and then significantly decreased when IL-6 levels were 30-fold higher than normal, which may indicate that these lymphocyte subsets play an essential role in anti-inflammatory function. Notably, the numbers of effector memory T4 cells, plasma blasts, and Tregs were remarkably higher with IL-6 levels were over 30-fold higher than normal, especially plasma blasts, which showed a positive correlation with IL-6 levels. It is known that effector memory T4 cells are reactivated by antigen exposure and expanded, which can protect the host [31]. Plasma blasts, also known as effector B cells, play a key role in synthesizing and storing antibodies and participating in humoral immune responses. Moreover, Tregs are also necessary for immunosuppression and participate in the occurrence and development of various immune diseases. Due to COVID-19 patients having a higher number of these three important lymphocyte subsets of effector memory T4 cells, plasma blasts, and Tregs, the levels of IL-6 were also significantly higher; thus, use of anti-IL-6 receptor (IL-6R) monoclonal antibodies (mAbs), such as tocilizumab therapy, may be effective for COVID-19 treatment [32, 33].

The immune system comprises a network of cells, tissues, and organs that mediate host defence against pathogens. Immune cells can be classified into distinct types based on specific surface markers. However, not all immune cell types can be completely addressed, as many cell markers are expressed by multiple cell lineages and regulated differently during inflammation. In recent years, sequencing technology has been widely used in biological research. In this study, scRNA-seq data were used to comprehensively characterize immune cell changes between COVID-19 patients and healthy individuals. Overall, lymphopenia was commonly found in COVID-19 patients compared to healthy controls, and both the highly expressed genes and subset-specific marker genes were significantly decreased in COVID-19 patients. These results are consistent with those reported by Wang et al. in COVID-19 patients in the recovery stage [34]. In addition, although the reduction in lymphocytes is clear, the changes in the functions of lymphocyte populations remain unknown. In the future, it will be necessary to test the functions of distinct immune cell populations to determine whether the innate or adaptive immunity is activated or impaired in COVID-19.

## Conclusion

In this study, our investigation indicates that the comprehensive decrease of in immune and lymphocyte subsets in critical patients with COVID-19 infection and peripheral lymphocyte subset alterations showed a clear association with clinical characteristics. Targeting inflammation such as IL-6 is a promising tool for the treatment of COVID-19 and provides evidence of the biological mechanism underlying the clearance of COVID-19.

## Supplementary Information

**Additional file 1**. **Figure S1** Representative flow cytometry dot plots showing the gating strategy for B lymphocyte subsets. **Figure S2** Representative flow cytometry dot plots showing the gating strategy for T lymphocyte subsets. **Figure S3** Representative flow cytometry dot plots showing the gating strategy for basic cell subsets. **Figure S4** Representative flow cytometry dot plots showing the gating strategy for Treg lymphocyte subsets.

## Data Availability

All data is available on request.

## Abbreviations

COVID-19: Coronavirus disease 2019
SARS-CoV-2: Severe acute respiratory syndrome coronavirus 2
IL-6: Interleukin-6
scRNA-seq: Single-cell RNA sequencing
NK: Natural killer
PBMC: Peripheral blood mononuclear cell
MERS-CoV: Middle East respiratory syndrome coronavirus
NKT: Natural killer T
Th: T helper
CTL: Cytotoxic T lymphocyte
mAb: Monoclonal antibody
