# Supplementary material for: Characteristics of lymphocyte subset alterations in COVID-19 patients with different levels of disease severity
Source: Virol J. 2022 Nov 19;19:192. doi: 10.1186/s12985-022-01926-8 (PMC9675966; doi:10.1186/s12985-022-01926-8)
Supplement: Supplementary file 1 — Additional file 1. Figure S1 Representative flow cytometry dot plots showing the gating strategy for B lymphocyte subsets. Figure S2 Representative flow cytometry dot plots showing the gating strategy for T lymphocyte subsets. Figure S3 Representative flow cytometry dot plots showing the gating strategy for basic cell subsets. Figure S4 Representative flow cytometry dot plots showing the gating strategy for Treg lymphocyte subsets. [file 12985_2022_1926_MOESM1_ESM.docx]

**Characteristics of lymphocyte subset alterations in COVID-19 patients** **with different levels of disease severity**

Supporting information


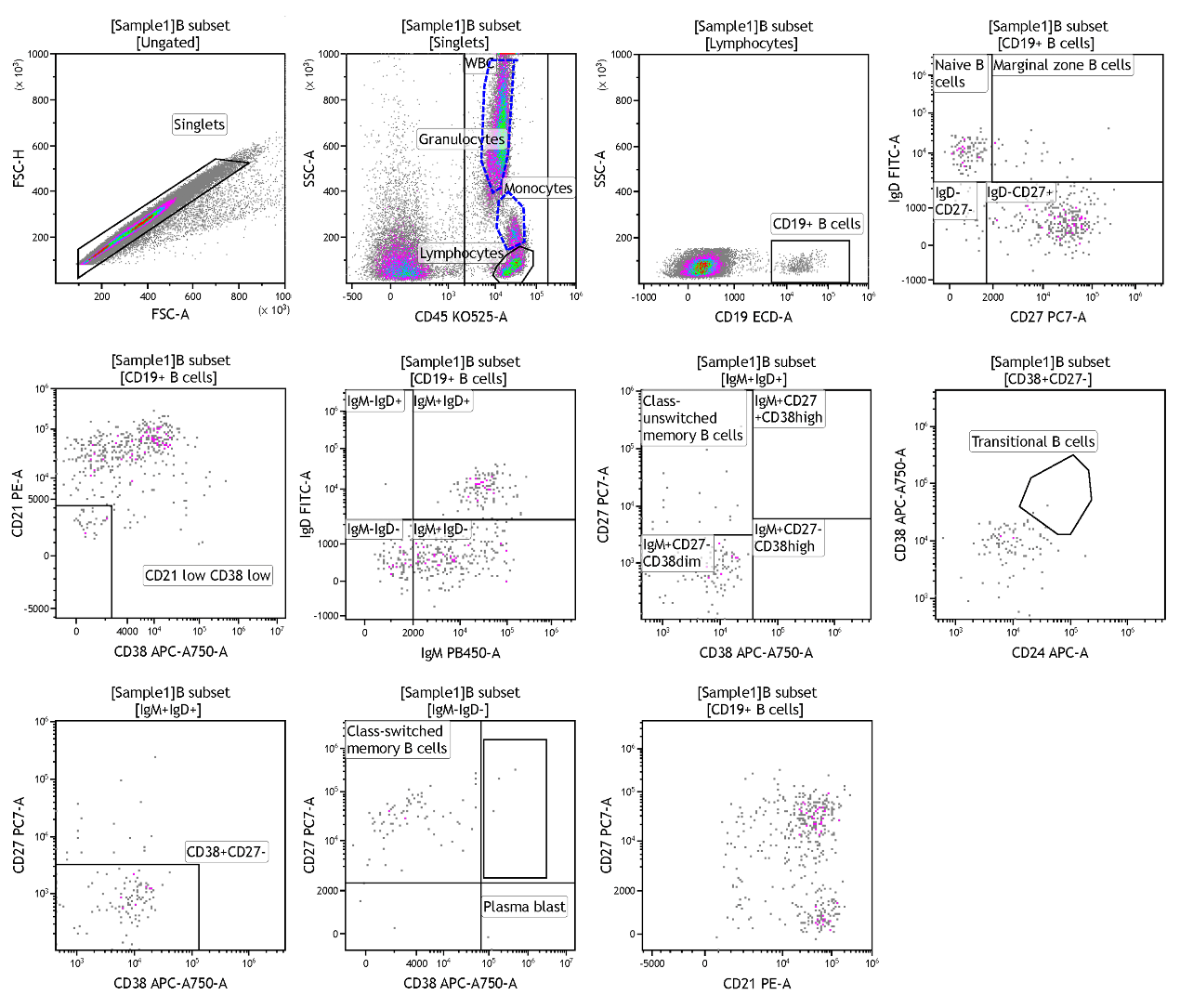


**Fig. S1** Representative flow cytometry dot plots showing the gating strategy for B lymphocyte subsets.


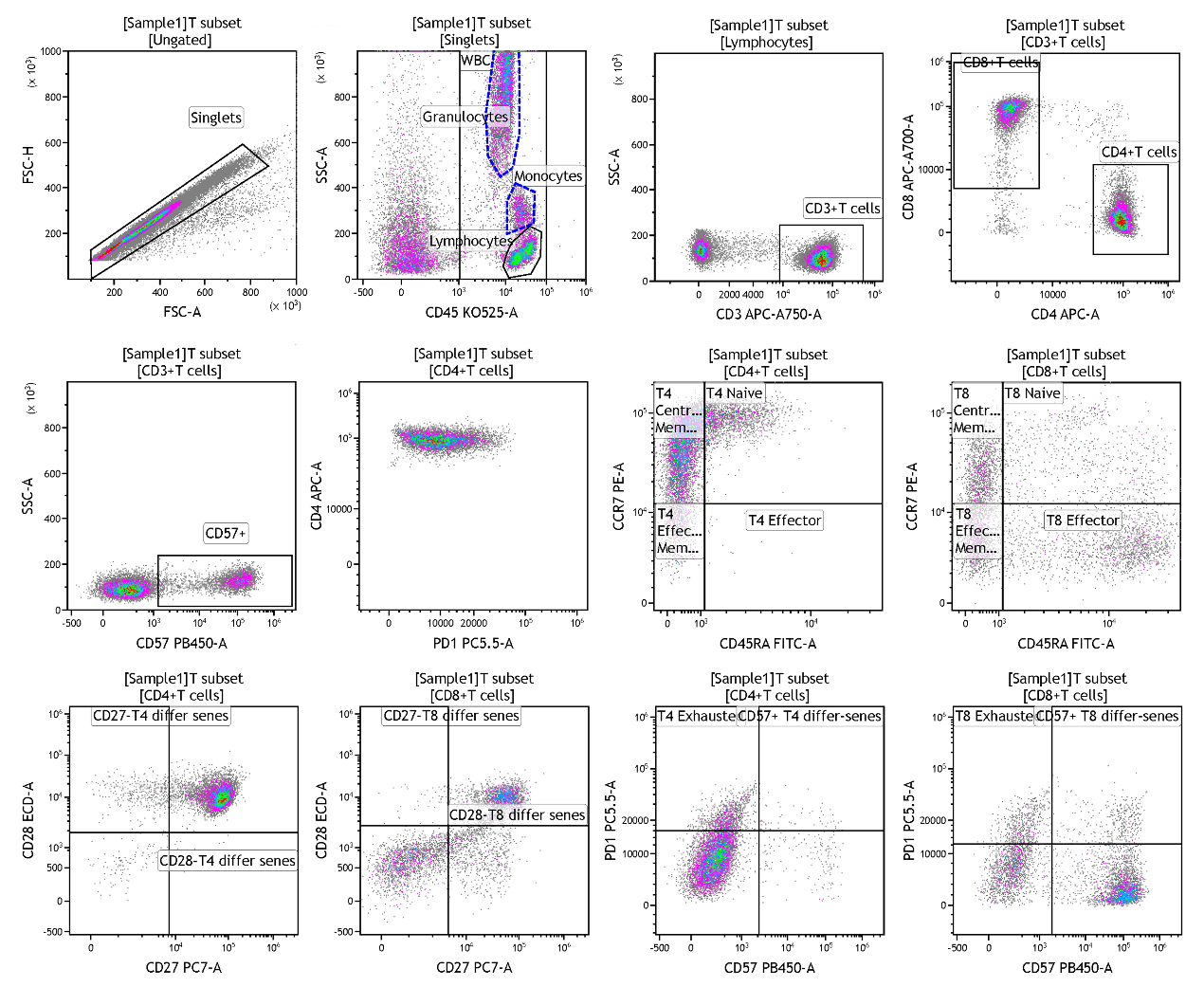


**Fig. S2** Representative flow cytometry dot plots showing the gating strategy for T lymphocyte subsets.


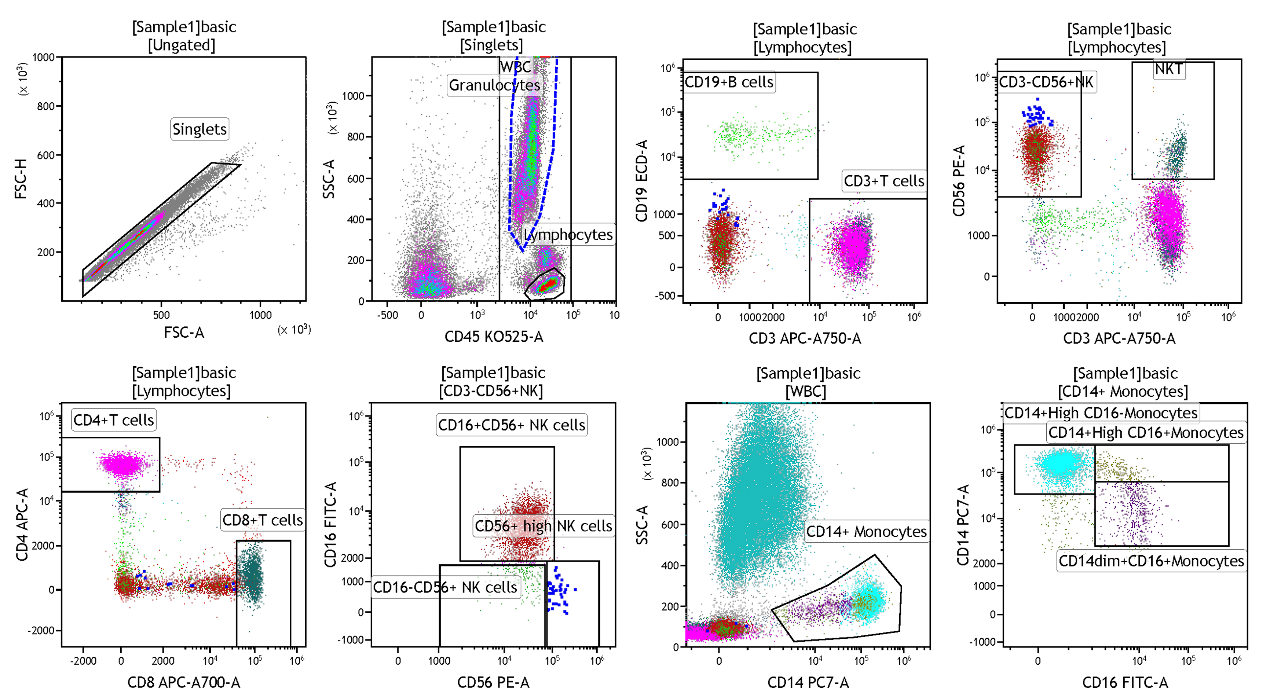


**Fig. S3** Representative flow cytometry dot plots showing the gating strategy for basic cell subsets.


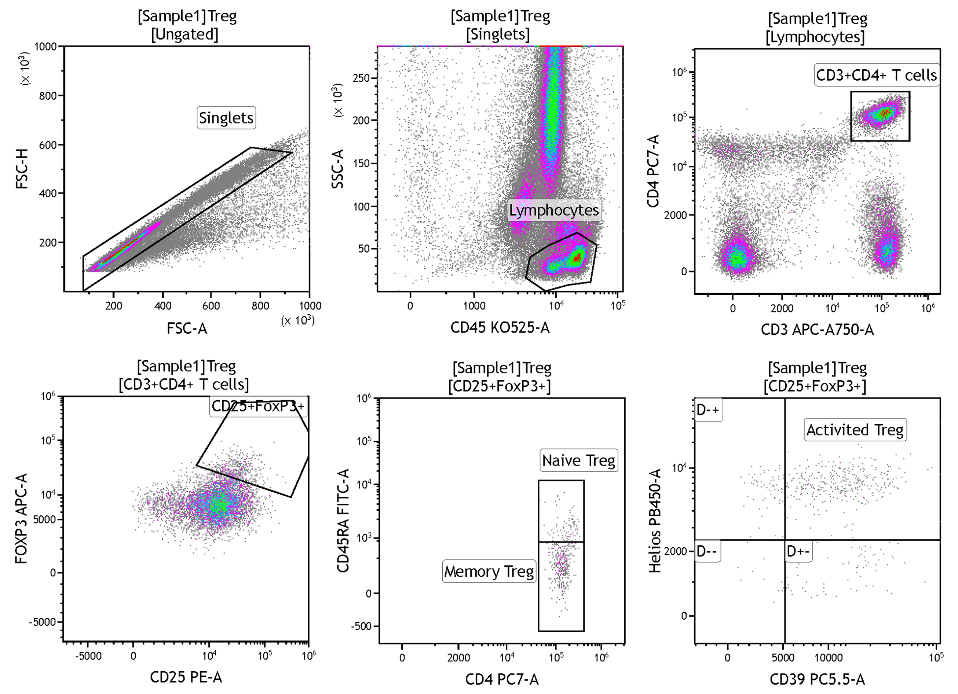


**Fig. S4** Representative flow cytometry dot plots showing the gating strategy for Treg lymphocyte subsets.
